# Functional Outcomes of Midshaft Clavicle Fractures Treated With Plate Osteosynthesis: A Prospective Observational Study

**DOI:** 10.7759/cureus.117209

**Published:** 2026-09-30

**Authors:** Brijendra Singh, Rohit Shukla, Shubham Srivastava, Ashutosh Yadav, Ajay Yadav

**Affiliations:** 1 Department of Orthopaedics, Balrampur Hospital Golaganj, Lucknow, IND; 2 Department of Orthopaedics, RKDF Medical College, Bhopal, IND; 3 Department of Orthopaedics, Institute of Medical Sciences, Banaras Hindu University, Varanasi, IND

**Keywords:** clavicle fracture, constant murley shoulder score (cms), fracture union, midshaft clavicle fracture, plate osteosynthesis

## Abstract

Introduction

Clavicle fractures are a common orthopedic injury and account for a significant proportion of shoulder girdle trauma, the majority involving the midshaft region. Although conservative treatment has traditionally been preferred, displaced fractures are often associated with complications such as non-union and functional impairment. Plate osteosynthesis has gained popularity because it provides stable fixation and allows early mobilization. This study aimed to evaluate the functional outcomes of patients with displaced midshaft clavicle fractures treated with plate osteosynthesis.

Methodology

A prospective observational study was conducted over one year in the Department of Orthopaedics, Balrampur Government Teaching Hospital, Lucknow, involving 30 adult patients with completely displaced midshaft clavicle fractures. All patients underwent open reduction and internal fixation with pre-contoured plates. Functional outcomes were assessed using the Constant-Murley shoulder (CMS) score at six weeks, 12 weeks, 18 weeks, and six months postoperatively. Fracture union was evaluated radiographically.

Results

The mean age was 41.57 years, with men constituting 73% of the cohort. Falls and road traffic accidents were the most common injury mechanisms. Fracture union was achieved in all cases at a mean duration of 12 weeks. The CMS score improved from a mean of 69.97 at six weeks to 88.97 at six months (repeated-measures ANOVA, F(3, 87) = 74.79, p < 0.0001). At six months, 50.00% (n = 15) of patients had an excellent outcome and 43.33% (n = 13) good outcome. Complications occurred in 20.00% (n = 6) of patients (infection 10.00%, delayed union 6.67%, and hardware failure 3.33%); 80.00% had no adverse events.

Conclusion

In this study, plate osteosynthesis for displaced midshaft clavicle fractures was associated with progressive functional improvement, a high union rate, and an acceptable complication profile. These findings describe outcomes in this cohort and do not establish comparative effectiveness; larger comparative studies with longer follow-up are needed.

## Introduction

The clavicle, or collarbone, forms a structural bridge between the sternum and scapula, making up the shoulder girdle. Due to its superficial location, it is prone to fractures, accounting for 2.6% to 4% of all adult fractures [1,2]. It is a common injury among young adults, with adolescent men accounting for more than one-third of clavicular fractures and adolescent women accounting for approximately one-fifth [3]. According to Allman’s classification [4], clavicle fractures are divided into three groups based on their anatomical location: middle-third, lateral-third, and medial-third fractures. Middle-third fractures are the most common, accounting for approximately 80% of all clavicle fractures, of which 48% are displaced [1,5]. Non-operative management has traditionally been preferred for most clavicle fractures; however, it has been associated with higher rates of non-union, malunion, and functional limitation, especially in displaced fractures [5,6]. Plate osteosynthesis is an established surgical option for selected clavicle fractures, particularly displaced and comminuted midshaft fractures, and may provide higher union rates and faster early functional recovery than non-operative treatment [6-8]. Operative fixation may also be considered in specific clinical situations, such as open fractures, associated neurovascular compromise, symptomatic non-union, and malunion with functional or neurovascular impairment [9,10]. Contemporary fixation methods, including pre-contoured anatomical plates and locking screw constructs, have further improved implant fit and fixation stability while potentially reducing implant-related complications [11]. Assessment of functional recovery remains important when evaluating the effectiveness of plate osteosynthesis, beyond radiographic evidence of fracture union. The Constant-Murley shoulder (CMS) score is a validated and widely used outcome measure that incorporates pain, activities of daily living, range of motion, and strength to provide a comprehensive assessment of shoulder function [12]. This study therefore aimed to evaluate functional outcomes in patients with displaced clavicle fractures treated with plate osteosynthesis using this score.

## Materials and methods

Study design and setting

This prospective observational study was conducted in the Department of Orthopaedics, Balrampur Government Teaching Hospital, Lucknow, India, from March 2024 to March 2025. Ethical clearance was obtained from the Institutional Ethics Committee (ref. no. SRC/2024/1032), and all procedures were performed in accordance with the Declaration of Helsinki. Written informed consent was obtained from all participants before enrollment. Patients were recruited using consecutive sampling, whereby all eligible patients presenting during the study period were included until the required sample size was achieved. This approach minimized selection bias and enhanced the external validity of the findings.

Sample size calculation

The sample size was calculated based on the following formula.

$N=Z^{2}\times p(1-p)/d^{2}$ 

$N = (1.96)^{2} &times; 0.766 &times; 0.234/(0.15)^{2}$

*N = 0.6886 / 0.0225 = 30.60*

where N = required sample size, Z = normal deviate at the portion of 95% confidence interval (CI) = 1.96, p = incidence of excellent functional outcome by using the Constant-Murley score of 76.6% (0.766), and d = margin of error at 15% (0.15). Considering a 95% confidence level, the calculated minimum sample size was 30.6, which was rounded up to 31. Only 30 eligible patients presented during the study period, and all were enrolled, one fewer than the calculated minimum.

Selection criteria

Patients aged ≥18 years with fresh (≤28 days from injury), displaced midshaft clavicle fractures (Allman's classification Group I) who were willing to undergo open reduction and internal fixation (ORIF) were included. Patients aged <18 years and those with undisplaced clavicle fractures, open or pathological fractures, associated neurovascular injuries, or polytrauma involving the ipsilateral upper limb or shoulder girdle were excluded, as these conditions could independently influence functional and radiological outcomes.

Preoperative assessment and surgical technique

Eligible patients underwent clinical and radiological evaluation. Fracture displacement was assessed on anteroposterior shoulder radiographs and defined as the absence of cortical contact between the proximal and distal fragments. Demographic characteristics, mechanism of injury, and associated injuries were recorded. Socioeconomic status was determined from occupation as documented in the outpatient questionnaire, without use of a validated socioeconomic scale, and categorized as low (daily-wage workers or manual laborers), middle (skilled workers, small traders, or salaried employees), or high (professionals or business owners).

All procedures were performed under general anesthesia by experienced orthopedic surgeons. Patients were positioned supine on a radiolucent table with a sandbag placed between the scapulae. A longitudinal oblique incision was made over the clavicle, centered on the fracture site. After exposure and reduction, fixation was achieved using a pre-contoured 3.5 mm locking compression plate placed on the superior surface of the clavicle with at least three screws securing each proximal and distal fragment. Large comminuted fragments were stabilized with lag screws while preserving their soft-tissue attachments. The deltotrapezial fascia was closed with interrupted absorbable sutures followed by skin closure. Operative duration and intraoperative blood loss measured by the standard intraoperative method were recorded.

Postoperative management and rehabilitation

Postoperatively, the limb was immobilized in an arm sling, and patients were advised to perform gentle pendulum exercises. Active shoulder mobilization was started at six weeks, when the sling was discontinued. Clinical and radiological follow-up was performed at six, 12, and 18 weeks and six months postoperatively.

Outcome measures

The primary outcome was functional recovery, assessed using the CMS score at each scheduled follow-up [12]. The CMS is a 100-point scale comprising pain (15 points), activities of daily living (20 points), range of motion (40 points), and strength (25 points). Pain and activities of daily living are patient-reported, whereas range of motion and strength are assessed clinically. Higher scores indicate better shoulder function, with scores of 90-100 considered excellent, 80-89 good, 70-79 fair, and <70 poor.

Secondary outcomes included time to radiological fracture union and the incidence of postoperative complications (delayed union, infection, and implant-related complications such as hardware failure). Fracture union was confirmed radiographically by complete cortical bridging across the fracture site between the proximal and distal fragments. Delayed union was defined as slower than expected radiographic healing with persistent visibility of the fracture line followed by subsequent evidence of progression toward union. Non-union was defined as the absence of progressive radiographic healing on serial follow-up.

Statistical analysis

Statistical analysis was performed using IBM SPSS Statistics for Windows, version 26.0 (IBM Corp., Armonk, NY, USA). Continuous variables are presented as mean ± standard deviation (SD) and categorical variables as frequency (percentage). As the Constant-Murley score was measured repeatedly in the same patients at six, 12, and 18 weeks and six months, changes over time were analyzed using repeated-measures ANOVA, followed by post hoc pairwise comparisons. A p-value < 0.05 was considered statistically significant.

## Results

The age distribution showed that 30% of patients were in the 38-47 years group, followed by 20% each in the 18-27 and 48-57 years groups. The 58-67 years group included 16.67%, while 13.33% were in the 28-37 years group. The mean age was 41.57 years with a SD of ±13.58 years (Figure 1).

**Figure 1:**
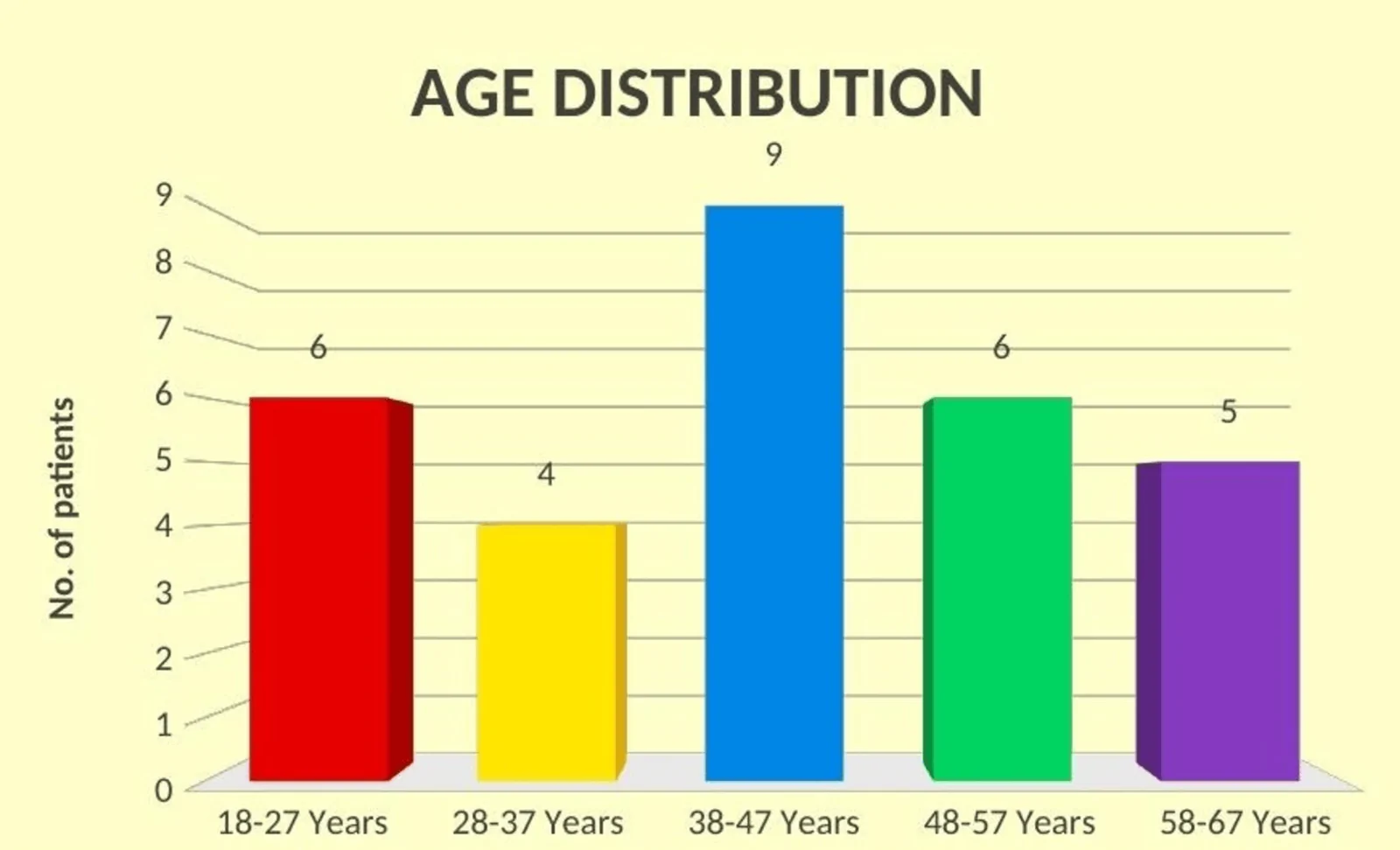
Age distribution of the enrolled patients.

Men comprised 73% of the sample, and women 27% (Figure 2).

**Figure 2:**
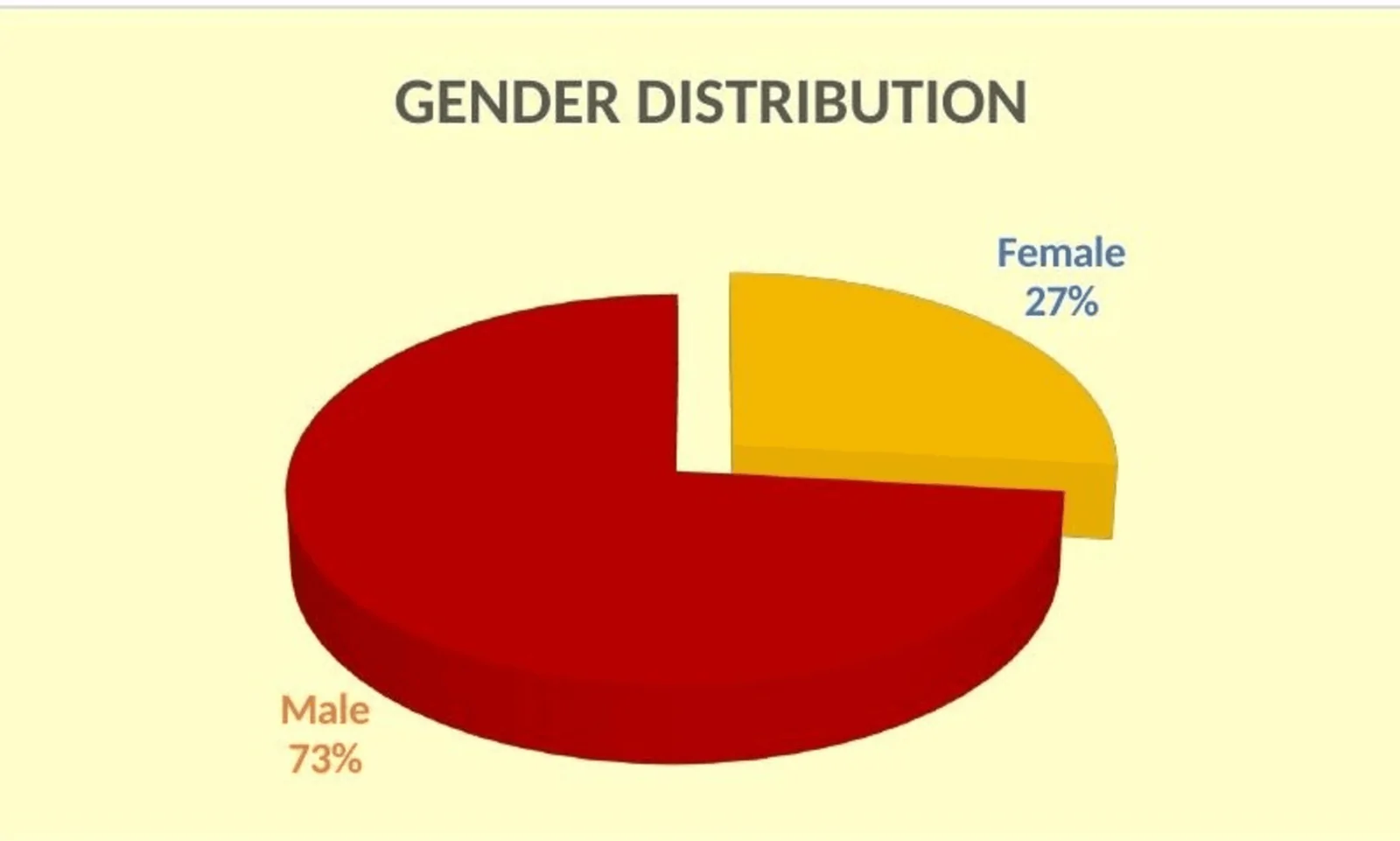
Gender distribution of the enrolled patients.

In terms of socioeconomic status, 53.33% of patients belonged to the middle-income group, 36.67% to the low-income group, and 10.00% to the high-income group (Figure 3).

**Figure 3:**
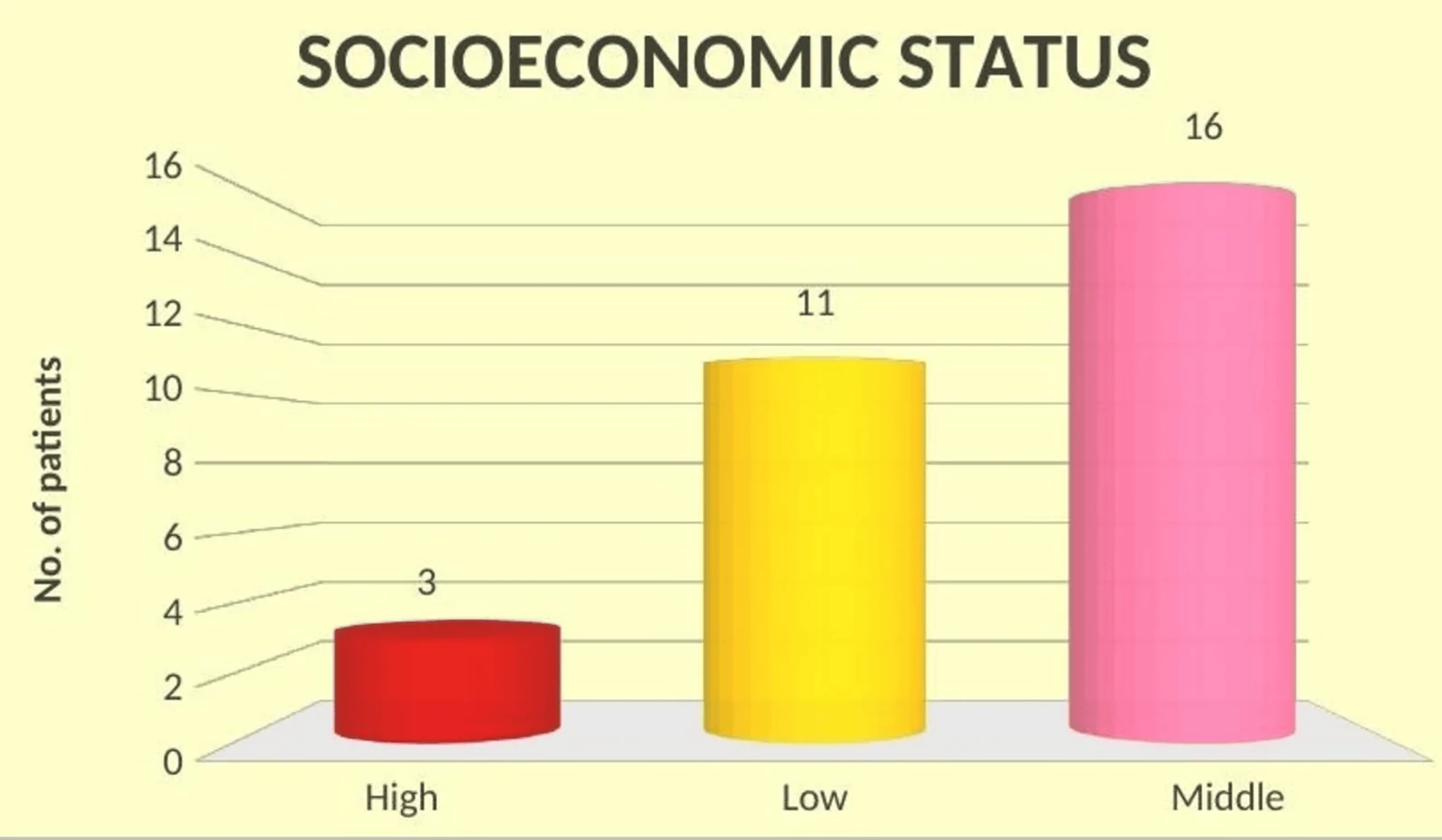
Socioeconomic status of the enrolled patients.

Falls and road traffic accidents (RTAs) were the leading causes of injury, each accounting for 43.33%, while sports injuries were responsible for 13.33% (Figure 4).

**Figure 4:**
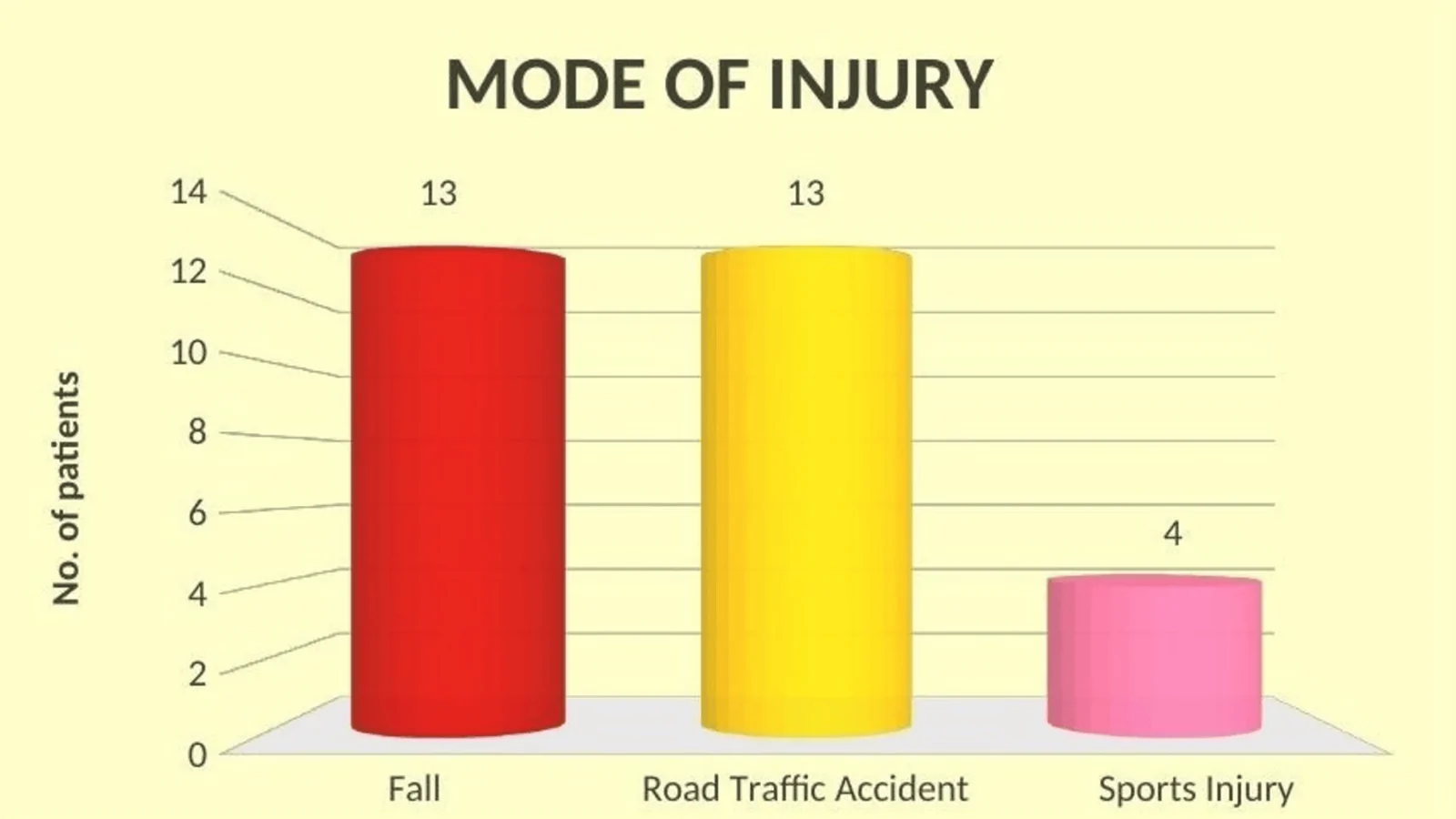
Mode of injury of the enrolled patients.

The mean fracture displacement was 15.23 mm (SD ±5.19). The average duration of surgery was 88.77 minutes, and the mean intraoperative blood loss was 179.00 mL. Fracture union occurred at a mean of 12.00 weeks (Table 1).

**Table 1:** Surgical parameters and healing metrics of the enrolled patients (N = 30). SD: standard deviation

Parameters	Mean	SD
Displacement (mm)	15.23	5.19
Surgery duration (min)	88.77	15.02
Blood loss (mL)	179.00	82.51
Time of union (weeks)	12.00	2.24

Functional outcomes assessed by the CMS score showed progressive improvement. The mean score increased from 69.97 ± 7.36 at six weeks to 78.83 ± 5.66 at 12 weeks, 84.30 ± 4.43 at 18 weeks, and 88.97 ± 5.80 at six months (repeated-measures ANOVA, F(3, 87) = 74.79, p < 0.0001), and all pairwise differences between time points were significant (Table 2). At six weeks, 53.33% of patients had poor outcomes, 36.67% fair, and 10.00% good. By six months, 50.00% had achieved excellent outcomes, 43.33% good, and 6.67% fair (Table 3).

**Table 2:** Constant-Murley shoulder score over follow-up periods. SD: standard deviation

Time point	Mean	SD
6 weeks	69.97	7.36
12 weeks	78.83	5.66
18 weeks	84.30	4.43
6 months	88.97	5.80

**Table 3:** Distribution of Constant-Murley shoulder score categories over time (N = 30). Categories based on the Constant-Murley shoulder score are defined as poor (<70), fair (70-79), good (80-89), and excellent (90-100). Data are presented as number (percentage). The same 30 patients were assessed at each time point; grades are presented descriptively.

Time point	Poor		Fair		Good		Excellent	
	n	%	n	%	n	%	n	%
6 weeks	16	53.33%	11	36.67%	3	10.00%	0	0.00%
12 weeks	3	10.00%	14	46.67%	12	40.00%	1	3.33%
18 weeks	0	0.00%	5	16.67%	21	70.00%	4	13.33%
6 months	0	0.00%	2	6.67%	13	43.33%	15	50.00%

Most patients (80.00%) experienced no complications. Delayed union was observed in 6.67%, hardware failure in 3.33%, and infection in 10.00% (Table 4).

**Table 4:** Complications observed among the enrolled patients.

Complication	Number	%
Delayed union	2	6.67%
Hardware failure	1	3.33%
Infection	3	10.00%
None	24	80.00%

## Discussion

In our study of 30 patients, the mean age was 41.57 years, with the highest proportion (30%) in the 38-47 years group. This contrasts with previous studies that report younger age groups. Fridberg et al. reported a median age of 36 years (range 14-75) in 105 patients treated with locking plate osteosynthesis [13]. Patel et al. found a mean age of 34 years (range 19-58) among 20 patients [14]. Karthi et al. noted most patients were below 30 years [15]. The gender distribution in our study showed 73% men and 27% women, consistent with literature reporting male predominance. Patel et al. observed 90% men and 10% women [14]. Karthi et al. reported 95% men and 5% women [15]. The higher incidence in men is linked to greater involvement in high-risk activities. Our slightly higher female proportion may reflect regional or demographic differences.

In our study, 53.33% of patients belonged to the middle socioeconomic group, 36.67% to the low group, and 10% to the high group. This pattern aligns with Patel et al., who observed a predominance of middle-income patients [14]. Karthi et al. also reported higher incidence among middle and lower socioeconomic groups [15]. The lower representation of high-income individuals may be due to better safety standards and reduced exposure to physical risk. Regarding the mode of injury, falls and RTAs each accounted for 43.33%, while sports injuries contributed 13.33%. This suggests high-impact trauma as the primary cause. Karthi et al. found RTAs in 60% of cases, followed by falls (30%) and sports injuries (10%) [15]. Dalal et al. reported RTAs at 48%, falls at 40%, and sports injuries at 12% [16]. Hathiwale et al. observed RTAs in 50%, falls in 35%, and sports injuries in 15% [17]. These findings are consistent with ours, highlighting the need for improved road safety and fall-prevention strategies.

The surgical and healing parameters in this study align with existing literature on plate osteosynthesis for clavicle fractures. The mean displacement of 15.23 mm (SD ±5.19) is consistent with Patel et al. and other studies reporting displacement of 14.6 to 16.2 mm, which support operative management for displacement greater than 15 mm [14-16]. The average surgery duration of 88.77 minutes (SD ±15.02) and blood loss of 179.00 mL (SD ±82.51) are comparable to Karthi et al. and Hathiwale et al. [15,17], and the mean union time of 12.00 weeks (SD ±2.24) reflects a predictable healing course. However, in the absence of a comparator group, these findings should not be interpreted as demonstrating superiority or causal effectiveness of plate fixation.

The CMS score improved progressively over the follow-up period (Table 2), consistent with previous reports on plate osteosynthesis for clavicle fractures. A mean score of 69.97 (SD ±7.36) at six weeks is in keeping with Patel et al. and Dalal et al. [14,16], and the score at 12 weeks (78.83, SD ±5.66) is similar to that reported by Karthi et al. [15]. The continued improvement to 84.30 (SD ±4.43) at 18 weeks and 88.97 (SD ±5.80) at six months is consistent with Hathiwale et al. [17]; Mishra et al. also reported better functional outcomes with surgical treatment than with conservative management [18]. The corresponding shift in Constant-Murley grades toward good and excellent outcomes over time (Table 3) mirrors the pattern reported by Patel et al., who found most patients reaching good to excellent scores by 12 weeks [14], and by Karthi et al., who noted improved scores over three to six months after plate osteosynthesis [15].

Complications occurred in 20.00% of patients (Table 4). This rate is at the upper end of, or slightly above, the range reported in previous studies: Patel et al. reported a 12% complication rate, including infection and non-union [14]; Karthi et al. observed complications in 15% of cases, predominantly infections [15]; Hathiwale et al. reported a 16% complication rate, with infection and delayed union the most common [17]; and Dalal et al. reported an 18% complication rate, mainly related to hardware [16]. These comparisons are descriptive, as definitions of complications, follow-up duration, and patient selection differed between studies, and the small sample of the present study gives a wide CI around our estimate (20.00%; 95% CI 7.7% to 38.6%). Plate osteosynthesis was therefore associated with an acceptable complication profile in this cohort, although comparative studies are needed to guide individualized treatment strategies.

## Conclusions

In this study, plate osteosynthesis for displaced midshaft clavicle fractures was associated with early mobilization, a high rate of union, and progressive functional improvement, along with an acceptable complication rate. These findings describe the course of recovery in this cohort and do not establish the comparative effectiveness of plate osteosynthesis. The study has several limitations, including a short follow-up of six months, a small sample, the absence of a control group, potential variability in rehabilitation adherence, and selection bias. Future research should therefore include longer follow-up, standardized rehabilitation protocols, and comparison with alternative and conservative treatments. Patient-reported outcomes and cost-effectiveness should also be evaluated to better define the role of surgical fixation in clavicle fracture management.
